# Ultra-Sensitive Nanoplatform for Detection of Brain-Derived Neurotrophic Factor Using Silica-Coated Gold Nanoparticles with Enzyme-Formed Quantum Dots

**DOI:** 10.3390/molecules30030699

**Published:** 2025-02-05

**Authors:** Seona Yu, Jaewon Choi, Yu-Rim Ahn, Minse Kim, Nanhyeon Kim, Hwunjae Lee, Hyun-Ouk Kim

**Affiliations:** 1 Division of Chemical Engineering and Bioengineering, College of Art, Culture and Engineering, Kangwon National University, Chuncheon-si 24341, Republic of Korea; 89seona@kangwon.ac.kr (S.Y.); choijw8839@kangwon.ac.kr (J.C.); reviana@kangwon.ac.kr (Y.-R.A.); minse0415@kangwon.ac.kr (M.K.); sksgus2000@kangwon.ac.kr (N.K.); 2 Department of Smart Health Science and Technology, Kangwon National University, Chuncheon-si 24341, Republic of Korea; 3 YUHS-KRIBB Medical Convergence Research Institute, Yonsei University College of Medicine, Seoul 03722, Republic of Korea; yohan0410@gmail.com; 4 Graduate Program of Biomedical Engineering, Yonsei University College of Medicine, Seoul 03722, Republic of Korea

**Keywords:** quantum dots (QDs), gold nanoparticles (GNPs), fluorescence detection, Alzheimer’s disease (AD)

## Abstract

A fluorescence-based detection platform was developed for brain-derived neurotrophic factor (BDNF), a key biomarker of Alzheimer’s disease (AD). This platform utilizes localized surface plasmon resonance effects resulting from the interactions between silica-coated gold nanoparticles (Au@SiO_2_) and enzymatically synthesized quantum dots (QDs). The gold nanoparticles were silica coated via the hydrolysis of tetraethyl orthosilicate, which allowed for precise control over the distance between the nanoparticles and QDs and refined the dynamics of fluorescence quenching and enhancement. Antibody conjugation was performed via sequential amination and carboxylation, followed by EDC/NHS coupling. BDNF was detected across a range of concentrations, from 1 ng/mL to 1 ng/mL, using an alkaline phosphatase (ALP)-conjugated polyclonal antibody targeting a secondary epitope of BDNF. The enzymatic hydrolysis of p-nitrophenyl phosphate by immobilized ALP led to the formation of cadmium sulfide QDs, with the fluorescence intensity correlating directly with the BDNF concentration. This platform offers a refined and precise method for detecting BDNF and is a reliable tool for the early diagnosis of AD.

## 1. Introduction

Alzheimer’s disease (AD) is the most common neurodegenerative disorder worldwide, accounting for approximately 65% of all cases of dementia [1,2]. It is characterized by progressive degeneration and the death of neurons, leading to memory impairment, cognitive dysfunction, and loss of independence in daily activities [3]. These features underscore that AD is not merely a consequence of aging but is driven by distinct pathological mechanisms. The hallmark pathology of AD includes the formation of two major lesions. First, amyloid beta (Aβ) peptides abnormally aggregate into extracellular plaques in the brain, disrupting neuronal communication and inducing inflammatory responses that lead to neuronal damage [4,5]. Second, the hyperphosphorylation of tau proteins destabilizes microtubules, forming intracellular neurofibrillary tangles that impair intracellular transport and ultimately induce neuronal death [6,7]. These pathological processes are interconnected, exacerbating the structural and functional deterioration of neuronal networks that manifests as memory loss and cognitive decline in the early stages [8].

The pathological changes in AD often begin several years before the emergence of clinical symptoms, emphasizing the need for reliable biomarkers for early diagnosis [9]. Established biomarkers for AD include Aβ42, tau proteins, and neurofilament light chains, the concentrations of which change with disease progression [10]. Additionally, brain-derived neurotrophic factor (BDNF) has emerged as a promising biomarker because of its critical role in neuronal survival, growth, and synaptic plasticity [11]. Early-stage AD often presents as mild cognitive impairment, and assessment using accurate biomarkers at this stage provide valuable opportunities to retard or manage disease progression [12]. However, current diagnostic methods, such as cerebrospinal fluid sampling and positron emission tomography imaging, are invasive, expensive, and complex, underscoring the need for reliable and non-invasive alternatives [13]. In this context, nano-biosensing technologies offer transformative potential by leveraging the pathological mechanisms and biomarker dynamics of AD. Platforms capable of quantitatively detecting neurotrophic factors such as BDNF can revolutionize the early diagnosis and monitoring of AD.

Nanoparticles play a pivotal role in advancing biosensor platforms because of their unique physicochemical and optical properties [14,15]. Gold nanoparticles (GNPs) are particularly notable for their excellent stability, biocompatibility, and localized surface plasmon resonance (LSPR) effects, which significantly enhance their sensitivity in biosensor applications [16,17]. Silica coating further improves GNP stability, reduces nonspecific interactions, and enables precise surface functionalization [18,19]. Quantum dots (QDs), known for their narrow emission spectra, high fluorescence quantum yield, and photostability, are also widely utilized in biosensing [20]. An enzyme-mediated synthesis of QDs, such as the formation of alkaline phosphatase (ALP)-catalyzed CdS QDs, offers a biocompatible and sustainable alternative to conventional methods, thereby improving safety and reproducibility [21].

**Scheme 1:**
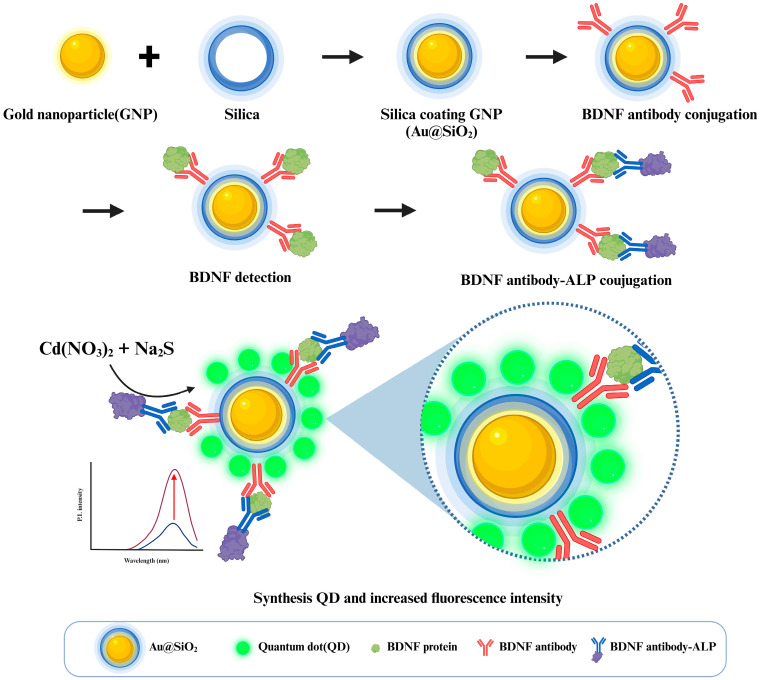
Schematic illustration of the ultra-sensitive detection platform for BDNF using Au@SiO_2_ and enzymatically synthesized QDs.

In this study, we aimed to integrate the unique properties of GNPs and QDs to develop a novel fluorescence-based nano-biosensing platform tailored to the early diagnosis of AD. Scheme 1 shows the whole process of the experiment. The proposed platform utilizes silica-coated GNPs (Au@SiO_2_) and enzymatically synthesized CdS QDs to detect BDNF. The silica coating on GNPs, achieved via tetraethyl orthosilicate (TEOS) hydrolysis and condensation, creates an optimal gap between the nanoparticles and QDs, enhancing the fluorescence amplification mechanism [22]. CdS QDs were synthesized using ALP, and their fluorescence intensity correlated directly with BDNF concentration. This platform addresses the limitations of existing invasive diagnostic methods, offering a sensitive and non-invasive alternative with the potential for multiplex biomarker detection. By leveraging the LSPR-induced fluorescence enhancement and precise spatial modulation, the proposed platform demonstrates significant promise as a next-generation diagnostic technology for AD and related neurodegenerative diseases.

## 2. Results and Discussion

### 2.1. Enzymatic Synthesis and Characterization of QDs

**Figure 1:**
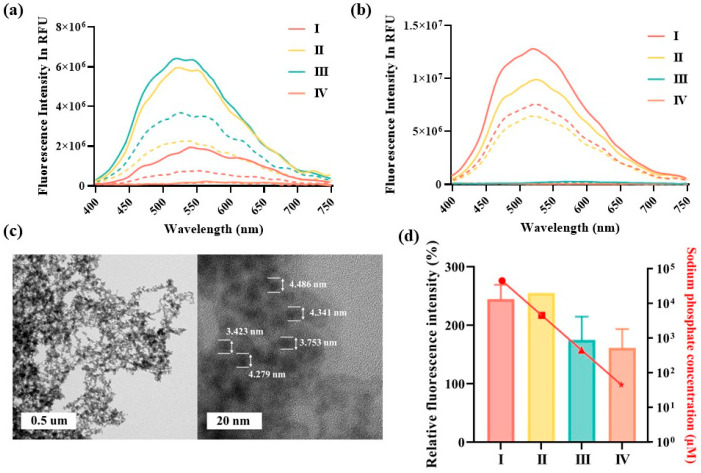
Analysis of QD formations under varying experimental conditions. (**a**) Comparison of the fluorescence intensities of QDs formed with different volumes of anti-mouse ALP (1 mg/mL): (I) 2 µL, (II) 4 µL, (III) 6 µL, and (IV) control (0 µL). Solid lines represent results from the incubation method and dashed lines represent results from the stirring method. (**b**) Comparison of the fluorescence intensities of QDs formed in the presence of different concentrations of sodium phosphate: (I) 45 mM, (II) 4.5 mM, (III) 450 µM, and (IV) 45 µM. Solid lines indicate results at 37 °C and dashed lines represent results obtained at room temperature. (**c**) TEM images displaying the morphology of QDs with a diameter of 4.056 nm (n = 5), confirming the uniformity and stability of the enzymatically synthesized QDs. (**d**) A quantitative analysis of QDs fluorescence intensity in the presence of different concentrations of sodium phosphate (I: 45 mM, II: 4.5 mM, III: 450 µM, IV: 45 µM) obtained using the ImageJ software. The fluorescence intensity (n = 10) is plotted as a linear graph, illustrating the relationship between sodium phosphate concentration and fluorescence intensity.

This enzymatic reaction utilizes p-nitrophenyl phosphate (pNPP) as the substrate, which is hydrolyzed by ALP to produce pNPP and orthophosphate ions [23]. These reaction products, upon interaction with Cd(NO_3_)_2_ and Na_2_S, facilitated the formation of stabilized CdS QDs. Preliminary experiments were conducted to confirm the independent synthesis of CdS QDs via enzymatic reactions prior to the BDNF screening. The fluorescence intensities of the synthesized QDs were compared to those of varying amounts of anti-mouse ALP under stirring and incubation conditions. Although stirring resulted in a lower overall fluorescence intensity than incubation, it displayed a more distinct dose-dependent trend, indicating enhanced enzyme–substrate interactions under stirring conditions (Figure 1a) [24]. Therefore, stirring was selected as the preferred method for subsequent experiments to ensure reliable and consistent QD formation. The fluorescence spectrum of the CdS QDs was further analyzed by substituting the orthophosphate ions with sodium phosphate as a stabilizing agent. Fluorescence intensity measurements at varying sodium phosphate concentrations at different temperatures revealed optimal fluorescence at specific concentrations, with lower concentrations showing significantly reduced fluorescence intensity and instability (Figure 1b). The higher fluorescence observed at 37 °C compared to that at room temperature highlights the temperature sensitivity of sodium phosphate as a substrate. At 37 °C, ALP exhibited optimal enzymatic activity under conditions closely resembling physiological environments, facilitating efficient hydrolysis of sodium phosphate and promoting robust QD synthesis [25]. Conversely, the reduced enzymatic activity at room temperature limited hydrolysis, leading to the incomplete formation of CdS QDs. These findings emphasize the dual importance of substrate concentration and temperature optimization for achieving an effective enzymatic synthesis. The morphological analysis of the CdS QDs synthesized via ALP-catalyzed reactions confirmed the successful formation of spherical particles with an average size of 4.056 nm, as determined using field emission transmission electron microscopy (FE-TEM) (Figure 1c). FE-TEM images provided direct visual evidence of particle formation, and individual particle sizes were determined using the ImageJ software, confirming their uniformity. This combination of FE-TEM and ImageJ analyses highlights the consistency and reproducibility of the enzymatic synthesis process, reflecting the stability of the reaction conditions between Cd(NO_3_)_2_ and Na_2_S. The fluorescence intensity was also quantitatively assessed across varying sodium phosphate concentrations using the ImageJ software (Figure 1d). Prominent fluorescence was observed at higher concentrations, whereas significantly diminished fluorescence was observed at lower concentrations, indicating that substrate availability is a critical factor for an efficient CdS QD synthesis. ImageJ analysis provided critical insights into the minimum fluorescence threshold required for reliable QD formation. This analysis reinforces the need for the precise optimization of substrate concentration and enzymatic reaction conditions to achieve consistent and reproducible results.

### 2.2. Characterization of GNPs and PEGylated GNPs

**Figure 2:**
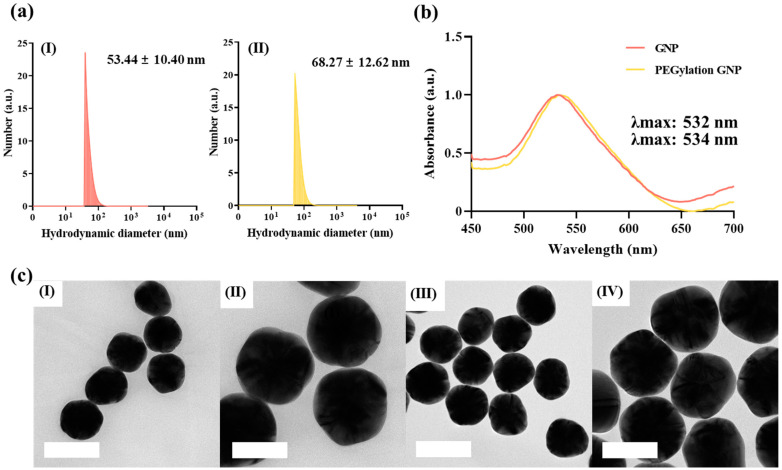
Characterization of bare and PEGylated GNPs. (**a**) The particle size distribution analyzed using dynamic light scattering. (I) The hydrodynamic diameter of unmodified GNPs was 53.44 ± 10.40 nm. (II) Increased hydrodynamic diameter of 68.27 ± 12.62 nm observed following PEGylation. (**b**) UV-vis absorption spectra displaying characteristic peaks for GNPs (λmax = 532 nm) and PEGylated GNPs (λmax = 534 nm), confirming successful surface modification. (**c**) TEM images illustrating the morphology of GNPs and PEGylated GNPs at varying magnifications. (I) GNPs when the scale bar was 200 nm. (II) GNPs when the scale bar was 100 nm. (III) PEGylated GNPs when the scale bar was 200 nm. (IV) PEGylated GNPs when the scale bar was 100 nm.

Uniform quasi-spherical GNPs were synthesized using the Tris-assisted premixing method, which offers a highly efficient and reproducible approach to achieving precise control over nanoparticle size and morphology [26]. A dynamic light scattering (DLS) analysis revealed that the synthesized GNPs had a hydrodynamic diameter of 53.44 ± 10.40 nm while PEGylated GNPs exhibited an increased diameter of 68.27 ± 12.62 nm (Figure 2a). This size increase indicated successful surface modification via PEGylation, reflecting the attachment of PEG molecules to the nanoparticle surface and the subsequent formation of a hydration layer [27]. The narrow size distribution observed in the PEGylated GNPs is indicative of uniform coverage by the PEG molecules. This is further supported by the minimized particle aggregation observed in DLS, confirming the effectiveness of PEGylation. The optical properties of the GNPs were also characterized, showing a distinct surface plasmon resonance (SPR) band at 532 nm, which shifted to 534 nm for the PEGylated GNPs. This red shift, depicted in the UV-visible absorption spectra (Figure 2b), indicates alterations in the surface properties of the GNPs following PEGylation which are attributed to changes in the refractive index and surface characteristics caused by the PEG attachment [28]. The observed red shift, driven by LSPR effects, highlights the sensitivity of nanoparticles to surface modifications and environmental changes, emphasizing the role of PEGylation in enhancing nanoparticle stability and modulating optical properties. A morphological analysis conducted using FE-TEM confirmed the spherical shape and uniform size of the synthesized GNPs. The FE-TEM images in Figure 2c show the distinct morphologies of the GNPs and PEGylated GNPs, demonstrating their spherical structure and uniformity at varying magnifications. In particular, the unmodified GNPs could be visualized when the scale bars were 200 nm (Figure 2c(I)) and 100 nm (Figure 2c(II)), whereas PEGylated GNPs retained a similar spherical morphology with scale bars of 200 nm (Figure 2c(III)) and 100 nm (Figure 2c(IV)). These findings validate the stability and reproducibility of the Tris-assisted premixing method. Collectively, these results confirm the successful synthesis of GNPs and their effective surface functionalization via PEGylation, underscoring the potential of the Tris-assisted premixing method for producing stable and functionalized nanoparticles.

### 2.3. Characterization of Au@SiO_2_ and Control of the Thickness of the Silica Coating

**Figure 3:**
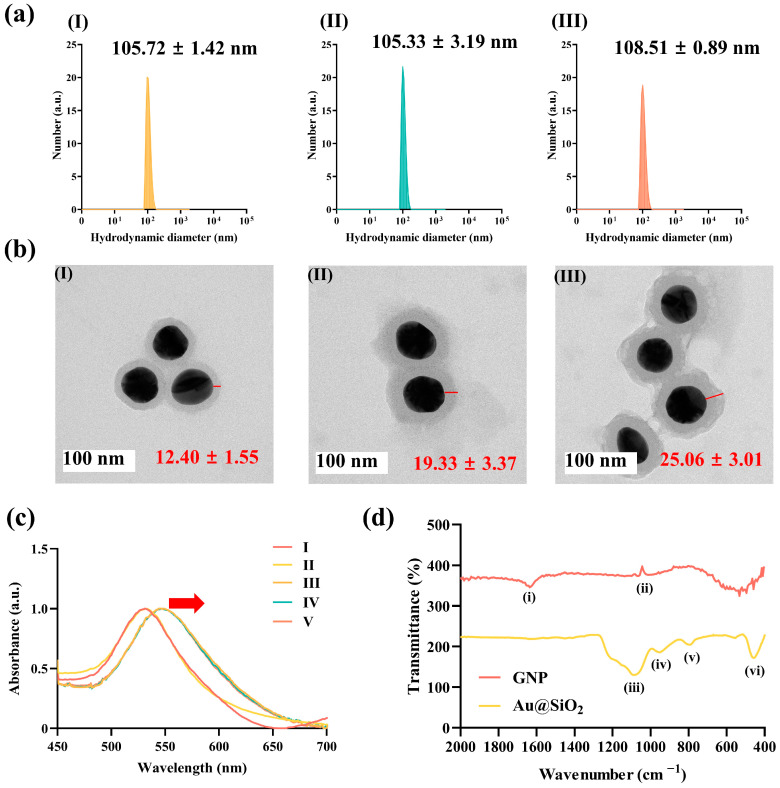
Comprehensive characterization of Au@SiO_2_ nanoparticles synthesized at varying TEOS concentrations. (**a**) A hydrodynamic diameter analyzed using dynamic light scattering (DLS) for Au@SiO_2_ synthesized with different concentrations of TEOS: (I) 10%, 105.72 ± 1.42 nm; (II) 30%, 105.33 ± 3.19 nm; (III) 50%, 108.51 ± 0.89 nm. (**b**) The morphological features and silica coating thickness observed using TEM: (I) TEOS 10%, 12.40 ± 1.55 nm; (II) TEOS 30%, 19.33 ± 3.37 nm; (III) TEOS 50%, 25.06 ± 3.01 nm. (**c**) UV-vis absorption spectra demonstrating a red shift with increasing TEOS concentrations: λmax = 544 nm (TEOS 10%), 547 nm (TEOS 30%), and 549 nm (TEOS 50%). (**d**) FT-IR spectra confirming the structural characteristics of GNP and Au@SiO_2_. Uncoated GNPs exhibited peaks for (i) C=O stretching (1635 cm^−1^) and (ii) C-N stretching (1043 cm^−1^), while Au@SiO_2_ showed peaks corresponding to (iii) Si-O-Si asymmetric stretching (1086 cm^−1^); (iv) Si-OH stretching (953 cm^−1^); (v) Si-O-Si symmetric stretching (793 cm^−1^); and (vi) Si-O bending (458 cm^−1^).

To modulate the spacing between the GNPs and QDs, Au@SiO_2_ was synthesized using a silica coating process based on the sol–gel method [29]. This approach involves the hydrolysis and condensation of TEOS, where TEOS reacted with water to form silanol (Si-OH) groups, which subsequently condensed to create Si-O-Si bonds [30]. The thickness of the silica coating was precisely controlled by varying the TEOS concentration to 10%, 30%, and 50%. A particle size analysis using DLS revealed mean hydrodynamic diameters of 105.72 ± 1.42 nm, 105.33 ± 3.19 nm, and 108.51 ± 0.89 nm for Au@SiO_2_ coated with 10%, 30%, and 50% TEOS, respectively (Figure 3a). These findings confirm the systematic increase in particle size corresponding to the gradual thickening of the silica layer. Prior to the application of the silica coating, the effect of the optical density (OD) of the GNPs on the coating process was examined systematically. Coatings were prepared at OD values of 1, 2, and 3, and an OD of 1 resulted in the formation of the most uniform and stable silica layers. Higher OD values of 2 and 3 caused residual silica deposits or excessive coating thicknesses, possibly owing to an increase in interparticle collisions, which disrupted the uniformity of the TEOS hydrolysis and condensation. Additionally, the reduced TEOS availability per particle, increased solution viscosity, diminished mixing efficiency, and altered local pH and ionic strength at higher OD values further hindered the formation of a consistent silica layer [31]. These results, supported by those of DLS, UV-vis absorbance, and FE-TEM analyses (Figure S1a–c), identified an OD of 1 as the optimal condition for achieving uniform coatings while minimizing interparticle interactions. The morphological properties of Au@SiO_2_ were characterized using FE-TEM. FE-TEM imaging confirmed the spherical morphology of the Au@SiO_2_ nanoparticles and revealed progressively thicker silica layers with increasing TEOS concentrations. ImageJ-based measurements indicated silica coating thicknesses of 12.40 ± 1.55 nm, 19.33 ± 3.37 nm, and 25.06 ± 3.01 nm for 10%, 30%, and 50% TEOS, respectively (Figure 3b). The increased thickness observed at higher TEOS concentrations was attributed to accelerated hydrolysis and condensation rates, with 50% TEOS leading to excessive silanol production and consequently, overcoating. These results demonstrated that the thickness of the silica coating can be precisely controlled by adjusting the TEOS concentration. The optical characteristics of Au@SiO_2_ were assessed using UV-vis absorbance measurements, which revealed a distinct red shift in the absorption spectrum compared to those of uncoated GNPs and PEGylated GNPs. This red shift, observed at 544, 547, and 549 nm for coatings with 10%, 30%, and 50% TEOS, respectively (Figure 3c), reflects changes in the refractive index induced by the silica layer and highlights the influence of the silica coating on the SPR properties of the GNPs. Fourier transform infrared (FT-IR) spectroscopy was used to confirm the structural integrity of the silica coatings [32]. For uncoated GNPs, characteristic peaks for C=O stretching at 1635 cm^−1^ and C-N stretching at 1043 cm^−1^ were observed. In contrast, Au@SiO_2_ exhibited distinct peaks corresponding to Si-O-Si asymmetric stretching at 1086 cm^−1^, Si-OH stretching at 953 cm^−1^, Si-O-Si symmetric stretching at 793 cm^−1^, and Si-O bending at 458 cm^−1^ (Figure 3d). These data validated the successful formation of silica layers on the GNP surfaces, thereby enhancing their structural stability and functionalization potential. To further evaluate the impact of the silica coatings, the relationship between the GNP and QD spacing was analyzed to optimize the fluorescence enhancement. The uniform and tunable silica coatings produced at OD = 1 and varying TEOS concentrations provide critical control over interparticle spacing, which is essential for maximizing the LSPR effects in biosensing applications. In summary, the successful synthesis of Au@SiO_2_ nanoparticles with a controllable silica coating thickness was achieved by optimizing the TEOS concentration and GNP OD values. An OD of 1 was determined to be ideal for achieving a uniform and stable coating. These findings reinforce the role of silica coatings in enhancing the performance of nanoparticle-based detection platforms by providing precise modulations for the interparticle spacing, ultimately contributing to improved fluorescence mechanisms in biosensing applications.

### 2.4. Mechanisms Underlying Fluorescence Quenching and Enhancement in Reactions with CdS QDs

**Figure 4:**
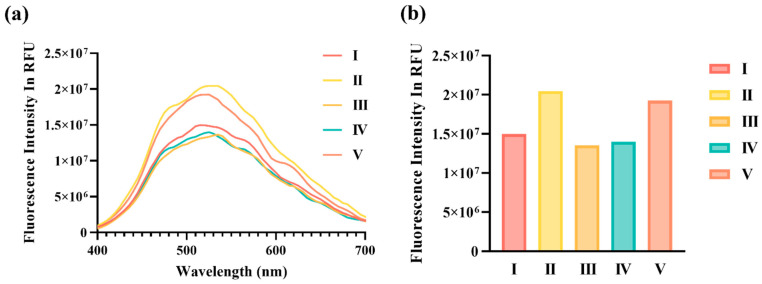
Fluorescence quenching and enhancement of QDs after interactions with GNPs and Au@SiO_2_ (varying TEOS concentrations). (**a**) Fluorescence emission spectra and (**b**) endpoint fluorescence intensities for QDs interacting with GNPs and Au@SiO_2_ synthesized using different TEOS concentrations. The data illustrate (I) GNPs showing fluorescence quenching due to direct interaction with QDs; (II) Au@SiO_2_ (TEOS 10%) exhibiting fluorescence enhancement attributed to optimal interparticle spacing; (III) Au@SiO_2_ (TEOS 30%) displaying reduced fluorescence enhancement; (IV) Au@SiO_2_ (TEOS 50%) showing fluorescence quenching caused by excessive interparticle spacing; (V) diluted QDs acting as the control.

To validate the fluorescence enhancement mechanism, the fluorescence intensities resulting from the interactions between GNPs, Au@SiO_2_, and CdS QDs were systematically compared. Control experiments were conducted using identical concentrations of GNP or Au@SiO_2_ to dilute CdS QDs under consistent experimental conditions. The interaction between the GNPs and CdS QDs resulted in a relatively low fluorescence intensity, indicating fluorescence quenching due to insufficient spacing between the particles. This lack of spacing suppresses the LSPR effect, limiting fluorescence enhancement (Figure 4a(I)) [22].

In contrast, a significant fluorescence enhancement was observed in the interaction between Au@SiO_2_ synthesized with 10% TEOS and CdS QDs. This result highlights that a silica coating thickness of 12.40 ± 1.55 nm, achieved at 10% TEOS, provides the optimal spacing between the CdS QDs and GNPs, effectively maximizing the LSPR effect (Figure 4a(II)). Supporting analyses, including FE-TEM imaging and ImageJ-based thickness measurements, confirmed that a precise interparticle distance is critical for fluorescence enhancement.

However, when the TEOS concentration was increased to 30% and 50%, leading to thicker silica coatings on Au@SiO_2_, fluorescence intensity declined, signaling a return to fluorescence quenching. At 30% TEOS, fluorescence enhancement was reduced (Figure 4a(III)), while, at 50% TEOS, the excessive silica thickness resulted in increased spacing between CdS QDs and Au@SiO_2_, diminishing the efficiency of the LSPR effect and causing fluorescence quenching (Figure 4a(IV)). This reduction in the fluorescence intensity is attributed to the weakened interaction of the localized electromagnetic field caused by the overly thick silica layer.

To explain the changes in fluorescence intensity as a function of the thickness of the silica coating, the following equation was used:

(1) $$I\left(d\right)=I_{0}\cdot \left(1+\eta \cdot \exp\left(-\frac{{\left(d-d_{opt}\right)}^{2}}{2\sigma^{2}}\right)\right)$$

where *I*(*d*) represents the fluorescence intensity at a silica coating thickness of *d*, *I*_0_ is the initial fluorescence intensity, *η* is the fluorescence enhancement factor determined by the LSPR effect, *d_opt_* is the optimal silica thickness, and *σ* denotes the standard deviation, indicating the effective range of enhancement. The experimental data were fitted to this equation to derive insights into the nonlinear effects of the silica coating thickness on fluorescence. The fluorescence intensity reached its maximum (*I*(*d*) = 2.0442 × 10^7^) at 10% TEOS, corresponding to the optimal thickness (*d_opt_* = 11.2 nm) at which the LSPR effect was maximized (Figure 4b(II)). Conversely, the fluorescence intensities for GNP (0% TEOS), 30% TEOS, and 50% TEOS were lower (1.4976 × 10^7^, 1.3536 × 10^7^, and 1.3997 × 10^7^, respectively), consistent with fluorescence quenching due to suboptimal spacing (Figure 4b(I,III,IV)). In summary, the ability to precisely control the thickness of the silica coating by adjusting the TEOS concentration was demonstrated to be essential for optimizing the spacing between GNPs and CdS QDs. This control is pivotal for achieving robust fluorescence enhancement, emphasizing the importance of interparticle spacing in the design of high-performance nanoparticle-based fluorescence platforms for biosensing applications.

### 2.5. Surface Modification for Antibody Conjugation on GNPs and Au@SiO_2_

**Scheme 2:**
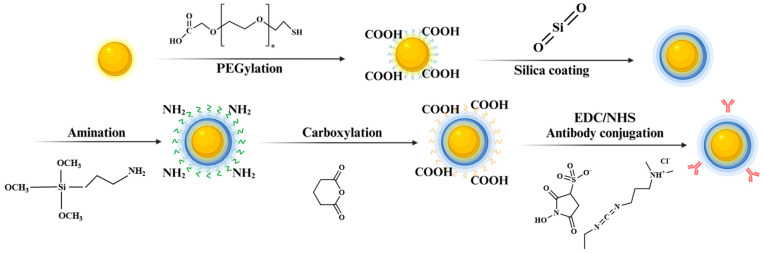
Illustration of the surface modification process for antibody conjugation.

**Figure 5:**
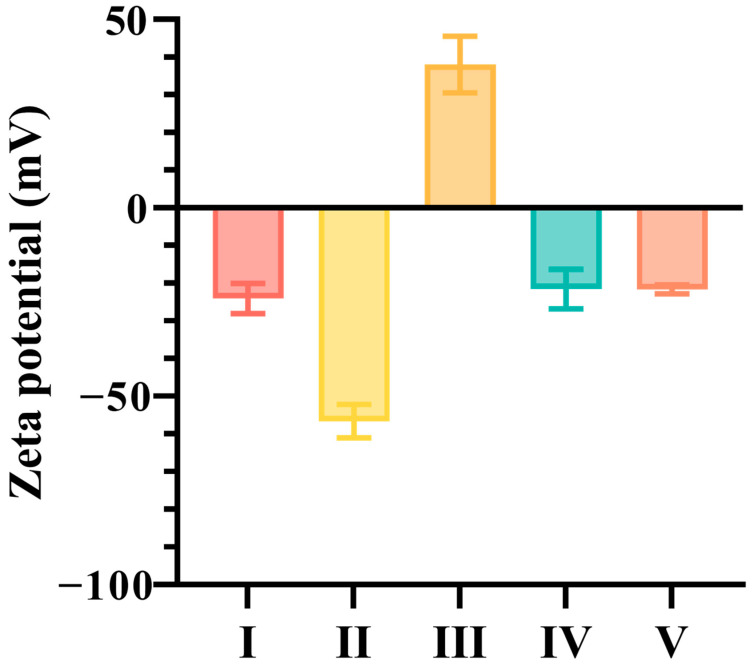
Analyzing the zeta potential at each step of the surface modification of silica-coated gold nanoparticles (TEOS 10%). Changes in the zeta potential for TEOS 10% Au@SiO_2_ across the following steps: (I) PEGylation of GNPs, (II) silica coating (Au@SiO_2_), (III) amine functionalization, (IV) carboxyl functionalization, and (V) antibody conjugation via EDC/NHS coupling.

The surfaces of the GNPs were systematically modified in a stepwise manner to enable anti-BDNF antibody conjugation, as depicted in Scheme 2. The modification process comprises PEGylation, silica coating, amination, carboxylation, and a final antibody conjugation via 1-ethyl-3-(-3-dimethylaminopropyl) carbodiimide hydrochloride (EDC)/N-hydroxysuccinimide (NHS) coupling chemistry. Zeta potential measurements were performed at each step to monitor surface charge variations, validate the success of the modifications, and compare the results with those of antibody-conjugated GNPs for control purposes (Figure S2). During the PEGylation step, the introduction of hydrophilic -OH groups by PEG molecules resulted in the formation of a hydration layer around the nanoparticles, which enhanced colloidal stability and prevented aggregation. This step resulted in a zeta potential of −24.05 ± 3.25 mV, indicative of stable particle dispersion. In the subsequent silica coating step, hydrolysis and condensation of TEOS produced Si-OH groups on the nanoparticle surface. These groups ionized to Si-O^−^ in aqueous environments, especially at a neutral to slightly basic pH, further reducing the zeta potential to −56.63 ± 3.61 mV (Figure 5(II)). This decrease highlighted the enhanced colloidal stability and formation of a reactive surface suitable for further functionalization. During the amination step, aminopropyltriethoxysilane (APTES) was used to introduce amine groups (–NH_2_) onto the nanoparticle surface. Under neutral or slightly acidic conditions, these groups protonated to form -NH_3_⁺, leading to a significant zeta potential shift to 38.08 ± 6.11 mV (Figure 5(III)). This positive charge confirms successful amination and provides the electrostatic interactions necessary for the subsequent functionalization steps. The carboxylation step involves the reaction of the amine-modified surface with succinic anhydride, which introduces carboxyl groups (-COOH). These groups ionized to -COO^−^ in aqueous solutions, resulting in a reduction in the zeta potential to −21.54 ± 4.32 mV (Figure 5(IV)). The negatively charged surface was then activated for covalent coupling with antibodies via EDC/NHS chemistry. The final step involved the covalent attachment of anti-BDNF antibodies to the carboxylated Au@SiO_2_ surface through EDC/NHS coupling. This process resulted in a slight increase in zeta potential to −21.64 ± 0.98 mV (Figure 5(V)), attributed to the partial neutralization of charges during amide bond formation [33]. This subtle shift confirmed the successful conjugation of the antibodies. As a control, antibody-conjugated GNPs were analyzed to ensure consistency and validity of the surface modification steps (Figure S2). Although the zeta potential of the control GNPs remained predominantly negative, reflecting their inherent surface properties, a slight increase was observed following antibody conjugation. This confirmed that the conjugation process was effectively applied across both Au@SiO_2_ and GNP platforms. The systematic variation in the zeta potential across the modification steps emphasizes the critical importance of each functionalization stage in optimizing nanoparticle surface properties for antibody conjugation. Together, the results presented in Scheme 2, Figure 5 and Figure S2 underline the precision required for surface engineering to enhance the antibody conjugation efficiency and improve biosensor performance.

### 2.6. Quantitative Analysis of BDNF Fluorescence Based on Concentration

**Figure 6:**
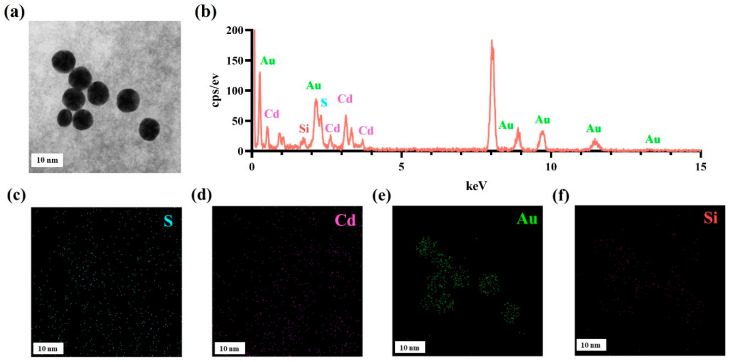
Confirmation of a QD formation around Au@SiO_2_ following BDNF detection via an elemental analysis. (**a**) TEM image showing BDNF detection in regard to Au@SiO_2_. ALP-conjugated antibodies specific to BDNF were attached, and QDs were formed through an enzymatic reaction on the nanoparticle surface. (**b**) An elemental ratio graph obtained from an EDS analysis showing the following weight percentages: Si 1.96%, S 10.72%, Cd 37.07%, and Au 50.25%. (**c**–**f**) Elemental distribution images showing the presence of (**c**) sulfur (S), (**d**) cadmium (Cd), (**e**) gold (Au), and (**f**) silicon (Si).

**Figure 7:**
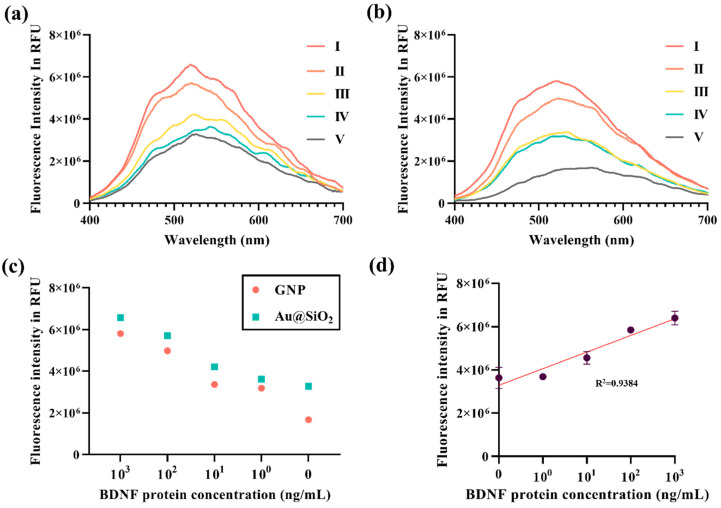
The quantitative detection of BDNF using GNPs and Au@SiO_2_. (**a**) Fluorescence intensity profiles for different concentrations of BDNF using Au@SiO_2_: (I) 1 μg/mL, (II) 100 ng/mL, (III) 10 ng/mL, (IV) 1 ng/mL, and (V) 0 ng/mL (Control). (**b**) Fluorescence intensity profiles for detecting BDNF using identical concentrations of GNP: (I) 1 μg/mL, (II) 100 ng/mL, (III) 10 ng/mL, (IV) 1 ng/mL, and (V) 0 ng/mL (control). (**c**) A comparative fluorescence detection analysis between GNPs and Au@SiO_2_ across varying BDNF concentrations, represented as endpoint relative fluorescence unit (RFU) values. (**d**) A linear correlation analysis of the endpoint fluorescence values for BDNF detection using Au@SiO_2_; a strong positive correlation with BDNF concentration was observed (R^2^ = 0.9384).

Enzymatic interactions between Au@SiO_2_ nanoparticles and CdS QDs demonstrated selective and efficient QD formation on nanoparticle surfaces. FE-TEM imaging confirmed the uniform assembly of CdS QDs facilitated by ALP-conjugated antibodies specific to BDNF (Figure 6a). An elemental composition analysis via EDS revealed the presence of Si (1.96%), S (10.72%), Cd (37.07%), and Au (50.25%) by weight (Figure 6b), validating the structural and compositional distribution of QDs on the Au@SiO_2_ surface. Spatial elemental mapping further highlighted the homogeneous distribution of S, Cd, Au, and Si (Figure 6c–f), emphasizing the precise interaction between the QDs and nanoparticles enabled by the enzymatic synthesis process. BDNF-mediated enzymatic reactions were further investigated using SEM and EDS analyses. In the absence of BDNF, SEM imaging showed minimal QD formation with extensive exposure of the Au@SiO_2_ surface (Figure S3(a-I)), while the EDS data indicated a predominance of Au (68.72%) with reduced Cd (26.93%) and S (4.35%) content (Figure S3(a-II)). In contrast, the presence of BDNF significantly enhanced QD formation, as evidenced by the SEM images (Figure S3(b-I)). An elemental analysis revealed a marked reduction in Au (3.56%) and a substantial increase in Cd (83.63%) and S (12.82%) levels (Figure S3(b-II)), confirming the role of BDNF-bound ALP antibodies in driving effective QD synthesis. Fluorescence detection using the Au@SiO_2_ platform demonstrated its high sensitivity across varying BDNF concentrations. Fluorescence intensity measurements for Au@SiO_2_ showed a gradual decline in fluorescence with a reduction in BDNF levels (Figure 7a), reflecting the platform’s responsiveness to concentration changes. In contrast, the GNP-based detection exhibited lower fluorescence intensity, which was attributed to the quenching effects arising from insufficient interparticle spacing (Figure 7b). A comparative analysis revealed the consistently superior performance of all concentrations of Au@SiO_2_ over that of GNPs (Figure 7c). In particular, Au@SiO_2_ exhibited fluorescence intensities 1.7 times higher than that of 1 μg/mL of GNPs and 1.9 times higher than that of 1 ng/mL of GNPs, highlighting the role of optimal interparticle spacing achieved via silica coating. A quantitative analysis confirmed a strong linear correlation between endpoint fluorescence intensity and BDNF concentration on Au@SiO_2_ (R^2^ = 0.9384; Figure 7d). This relationship underscores the precision and reliability of the platform for detecting and quantifying BDNF. The role of the silica coating in optimizing the spacing between the CdS QDs and GNP by enhancing the LSPR was pivotal in achieving fluorescence amplification. These findings demonstrated the importance of controlling the interparticle distances in fluorescence-based detection platforms. By leveraging precise enzymatic interactions and optimized nanoparticle designs, the Au@SiO_2_ platform presents a robust and sensitive tool for biomarker detection, advancing its potential for diagnostic applications.

## 3. Materials and Methods

### 3.1. Materials

Gold(III) chloride trihydrate (≥99.9% trace metals basis, HAuCl_4_·3H_2_O), silver nitrate (ReagentPlus^®^, ≥99.0% pure (titration) AgNO_3_), Tris(hydroxymethyl)aminomethane, trisodium citrate dihydrate (Na_3_C_6_H_5_O_7_, 99% pure), ammonium hydroxide solution (30–33% pure NH_3_ in H_2_O), TEOS, 3-[2-(2-aminoethylamino)ethylamino]propyl-trimethoxysilane (3-APTMS), glutaric anhydride (95%), N,N-dimethylformamide, (2-(N-morpholino)ethanesulfonic acid) [MES, low moisture content, 99% (titration)], N-(3-dimethylaminopropyl)-N′-ethylcarbodiimide hydrochloride, crystalline EDC, NHS, bovine serum albumin (BSA), anhydrous magnesium chloride (≥98% pure), pNPP, disodium salt hexahydrate (≥99% pure in HPLC, crystalline ALP-based ELISA assay substrate, C_6_H_4_NO_6_P · 2Na·6H_2_O; Calbiochem^®^), Na_2_S, and cadmium nitrate tetrahydrate (99.997% pure trace metals basis, Cd(NO_3_)_2_·4H_2_O) were purchased from Sigma–Aldrich (St. Louis, MO, USA). CM-PEG-SH-3400 was purchased from Laysan Bio (Arab, AL, USA), ethyl alcohol was purchased from Daejung Chemical Co., (Siheung-si, Republic of Korea), phosphate-buffered saline (PBS) was purchased from LPS Solution Co., (Daejeon, Republic of Korea), BDNF polyclonal antibody (PA1-18371) was purchased from Thermo Fisher Scientific Inc., Tween 20 was purchased from GenDEPOT, recombinant human BDNF protein (ab206642) was purchased from Abcam (Cambridge, UK), BDNF antibody (N-terminal; ALP-conjugated) was purchased from antibodiesonline (Pennsylvania, PA, USA), and Tris(2-carboxyethyl)phosphine hydrochloride was purchased from TCI (Tokyo, Japan).

### 3.2. Forming GNPs in the Size Range of 60 to 70 nm

GNPs were synthesized using a Tris-assisted premixing method, as outlined in the following protocol. A 0.5 mL aqueous solution of 1 wt% HAuCl_4_ was reacted with 42.5 µL of 0.1 wt% AgNO_3_ under stirring at 400 rpm. Subsequently, 0.4 mL of a 1 wt% citrate solution was added to the mixture while stirring, and distilled water (DW) was added to bring the total volume to 1.5 mL. The reaction mixture was incubated for 4 min to stabilize the nascent AuNPs. After the initial reaction, 2 mL of 0.1 M Tris buffer (pH 9.0) was introduced under stirring, followed by 1 min of reaction time. The solution was then rapidly injected into 47.5 mL of DW at 97 °C and subjected to vigorous stirring for 30 min. A visible color change from transparent pink to deep red was observed within the first minute, indicating the formation of AuNPs, with the color intensifying over the reaction period. The reaction mixture was allowed to cool to room temperature and then centrifuged at 10,000 rpm for 20 min to purify the nanoparticles. The resulting AuNPs were diluted to an OD of 1.0 using a microplate reader, targeting their characteristic absorbance peak at 535 nm, indicative of the formation of the AuNPs of the 60 nm diameter. The particle size distribution was analyzed using DLS, and the morphological characteristics were confirmed using FE-TEM.

### 3.3. PEGylation of GNPs

A PEGylation process was used to enhance the stability of GNPs. A 400 µL solution of 0.1 mM carboxymethyl-PEG-thiol (CM-PEG-SH; MW, 3400) was added to 6 mL of AuNPs under stirring at 1200 rpm. The reaction was allowed to proceed for 2 h to ensure efficient surface functionalization. The resulting PEGylated GNPs were purified via centrifugation at 10,000 rpm for 20 min, followed by washing to remove unreacted components. The purified nanoparticles were then dispersed in DW and refrigerated to maintain their stability. DLS was used to assess the dispersion quality, and FE-TEM was used to visualize the morphology and confirm the uniformity.

### 3.4. Coating a GNP Surface with Silica Precursor

To prepare Au@SiO_2_, 200 µL of ammonia solution was added to 20 mL of ethanol under continuous stirring at 400 rpm. A 3 mL aliquot of PEGylated GNP solution and 100 µL of TEOS were then introduced into the reaction mixture. TEOS was prediluted in ethanol, and its concentration was adjusted to 10%, 30%, and 50% to control the thickness of the silica layer. The reaction was allowed to proceed for 24 h to ensure complete coating. After the reaction, the samples were washed twice to remove the unreacted materials and subsequently dispersed in DW. The particle size of the resulting Au@SiO_2_ was measured using DLS. The silica coating was confirmed using FE-TEM imaging, and the thickness of the silica layer, which varied according to the TEOS concentration, was quantified using the ImageJ software. Additionally, the silica coating-induced red shift in the absorption spectra was analyzed using a microplate reader.

### 3.5. Modification of the Amine Group of Au@SiO_2_

Au@SiO_2_ dispersed in ethanol (6 mL) were functionalized by adding 40 µL of 3-APTMS and 40 µL of ammonia solution. The mixture was stirred at 1200 rpm for 1 h to introduce amino groups onto the nanoparticle surface. After the reaction, the unreacted materials were removed by washing the nanoparticles with a 1:1 mixture of DW and ethanol at 13,000 rpm for 20 min. The surface modification was verified by measuring the zeta potential, which indicated changes in the surface charge due to the successful incorporation of amino groups. This process ensures precise functionalization for further applications.

### 3.6. Carboxyl Group Modification of Amine Group-Modified Au@SiO_2_

A solution of glutaric anhydride (5 mM) was prepared in dimethyl formamide and stirred at 1200 rpm for 2 h to ensure complete dispersion. The pellet of aminated Au@SiO_2_ nanoparticles was subsequently resuspended in this solution. The reaction mixture was stirred for 24 h to facilitate the carboxyl group modification of the nanoparticle surface. After the reaction, the modified nanoparticles were washed three times by centrifugation at 13,000 rpm for 30 min to remove unreacted residues. Surface modification was confirmed by measuring the zeta potential, which verified the successful introduction of carboxyl groups onto the nanoparticle surface.

### 3.7. Antibody Conjugation via EDC/NHS Coupling

To conjugate a primary antibody to carboxylated Au@SiO_2_, an EDC/sulfo-NHS coupling reaction was used. In particular, 1 mL of Au@SiO_2_-COOH nanoparticles was mixed with 50 µL MES buffer (0.1 M, pH 5.5), 5 µL EDC (5 mg/mL), and 5 µL sulfo-NHS (5 mg/mL). The mixture was stirred at 200 rpm for 30 min to activate the carboxyl groups. Following activation, the nanoparticles were washed once via centrifugation at 13,000 rpm for 20 min and resuspended in DI water. The EDC/sulfo-NHS-functionalized solution was further incubated with 100 µL PBS (120 mM) and 20 µg of antibody for 1 h under stirring. To block nonspecific binding sites, 50 µL of a 10 wt% BSA solution was added and incubated for an additional 30 min. The resulting nanoparticles were washed twice with 20 mM PBS at 4 °C to remove unbound reagents. Finally, the antibody-conjugated Au@SiO_2_ was dispersed in 1 mL of 20 mM PBS containing 0.5% BSA and stored at 4 °C.

### 3.8. Fluorescence-Based Detection of BDNF via ALP-Mediated QD Formations

To screen the concentration of BDNF protein, 40 µL of antibody-conjugated Au@SiO_2_ nanoparticles were incubated with 100 µL of BDNF protein solution (prepared in 20 mM PBS, pH 8.0) at concentrations of 1 µg/mL, 100 ng/mL, 10 ng/mL, and 1 ng/mL for 1 h. After incubation, the samples were washed three times with 200 µL of PBS containing 0.05% Tween 20 (PBST) to remove the unbound proteins. For introducing the secondary antibody, 2 µg of anti-mouse ALP-conjugated antibody was added to the samples and incubated for 1 h at room temperature using a rotator. The samples were then washed three times with PBST and, finally, with Tris-HCl buffer (50 mM, pH 8.8) containing 1 mM MgCl_2_ to stabilize the complexes. To induce and stabilize the formation of CdS QDs, ALP activity was initiated using 90 µL of a substrate solution containing 0.56 mM pNPP in Tris-HCl buffer (50 mM, pH 8.8) supplemented with MgCl_2_. The mixture was incubated for 30 min at 37 °C. Subsequently, 5 µL of 6 mM sodium sulfide and 10 µL of 12.5 mM cadmium nitrate tetrahydrate were added to the samples, followed by an additional incubation for 30 min to facilitate QD formation. The fluorescence intensity of the QDs, which reflects the concentration of the detected BDNF protein, was measured using a microplate reader at an excitation wavelength of 290 nm. These fluorescence measurements enabled the quantitative analysis of BDNF concentrations based on the enzymatic activity of ALP and the corresponding QD formation.

## 4. Conclusions

A fluorescence-based BDNF detection platform was developed using Au@SiO_2_ nanoparticles in conjunction with enzymatically synthesized CdS QDs. The sensitivity and fluorescence amplification achieved with Au@SiO_2_ were higher than those of GNPs. The silica coating on Au@SiO_2_ provided an optimal spatial arrangement between the nanoparticles and CdS QDs, facilitating a more efficient fluorescence enhancement mechanism and enabling the precise quantitative detection of BDNF at varying concentrations. The experimental findings revealed that Au@SiO_2_ consistently exhibited a significantly higher fluorescence intensity than GNPs, even at the lowest concentration tested (1 ng/mL). In particular, Au@SiO_2_ demonstrated an approximately 1.9 times greater sensitivity at low concentrations, establishing its efficacy in detecting biomarkers in minimal quantities. A strong linear relationship (R^2^ = 0.9384) between the fluorescence intensity and BDNF concentration further validated the utility of the platform as a reliable quantitative diagnostic system. Fluorescence measurements were conducted using a microplate reader alongside TEM-EDS analyses, which confirmed the selective formation of CdS QDs on the Au@SiO_2_ surface, with fluorescence signals responding sensitively to the presence of BDNF. This selective localization of the CdS QDs contributed significantly to the increased sensitivity and reliability of the detection system. Compared with GNP-based platforms, Au@SiO_2_ effectively mitigated fluorescence quenching and maximized fluorescence enhancement, thereby delivering a robust and efficient detection mechanism. The findings of this study underscore the importance of the surface modification of Au@SiO_2_ for improving the performance of biosensing platforms. The developed platform demonstrated considerable promise as a next-generation diagnostic tool for the early detection and quantitative measurement of biomarkers, including BDNF, which are closely associated with neurodegenerative disorders. Future research should focus on extending the application of the platform to additional biomarkers and evaluating its feasibility in clinical diagnostics, thereby broadening its potential use. In conclusion, the Au@SiO_2_-based fluorescence detection platform introduced in this study represents a significant advancement in biomarker detection technologies, offering unparalleled sensitivity, reliability, and capability in regard to detecting biomarkers at trace levels.

## Data Availability

Data are contained within the article and Supplementary Materials.

## Supplementary Materials

The following supporting information can be downloaded at: https://www.mdpi.com/article/10.3390/molecules30030699/s1. Figure S1: Analysis of the DLS size distribution, UV-vis absorption spectra, and TEM images of GNPs and Au@SiO_2_ at varying optical densities (OD). Figure S2: Analysis of the zeta potential of antibody-conjugated GNPs as a control. Figure S3. Validation of quantum dot formation around Au@SiO_2_ using an elemental analysis following BDNF detection.
