# Supplementary material for: Ultra-Sensitive Nanoplatform for Detection of Brain-Derived Neurotrophic Factor Using Silica-Coated Gold Nanoparticles with Enzyme-Formed Quantum Dots
Source: Molecules. 2025 Feb 5;30(3):699. doi: 10.3390/molecules30030699 (PMC11820963; doi:10.3390/molecules30030699)
Supplement: Supplementary file 1 [file molecules-30-00699-s001.zip › molecules-3426307-supplementary.pdf]

Supporting Information

Ultra-sensitive Nanoplatform for Detection of Brain-derived Neurotrophic Factor using Silica-coated Gold Nanoparticles with Enzyme-formed Quantum Dots

Seona Yu [1,2 †], Jaewon Choi [1,2†], Yurim Ahn[1,2], Minse Kim [1,2,] Nanhyeon Kim [1,2], Hyunjae Lee [3,4] and Hyun-Ouk Kim [1,2,*]

[1] Division of Chemical Engineering and Bioengineering, College of Art, Culture and Engineering, Kangwon National University, Chuncheon-si, Gangwon-do 24341, Republic of Korea

[2] Department of Smart Health Science and Technology, Kangwon National University, Chuncheon 24341, Republic of Korea

[3] YUHS-KRIBB Medical Convergence Research Institute, Yonsei University College of Medicine

[4] Graduate Program of Biomedical Engineering, Yonsei University College of Medicine

*Correspondence: kimhoman@kangwon.ac.kr (H.O.)

‡ These authors contributed equally to this work.

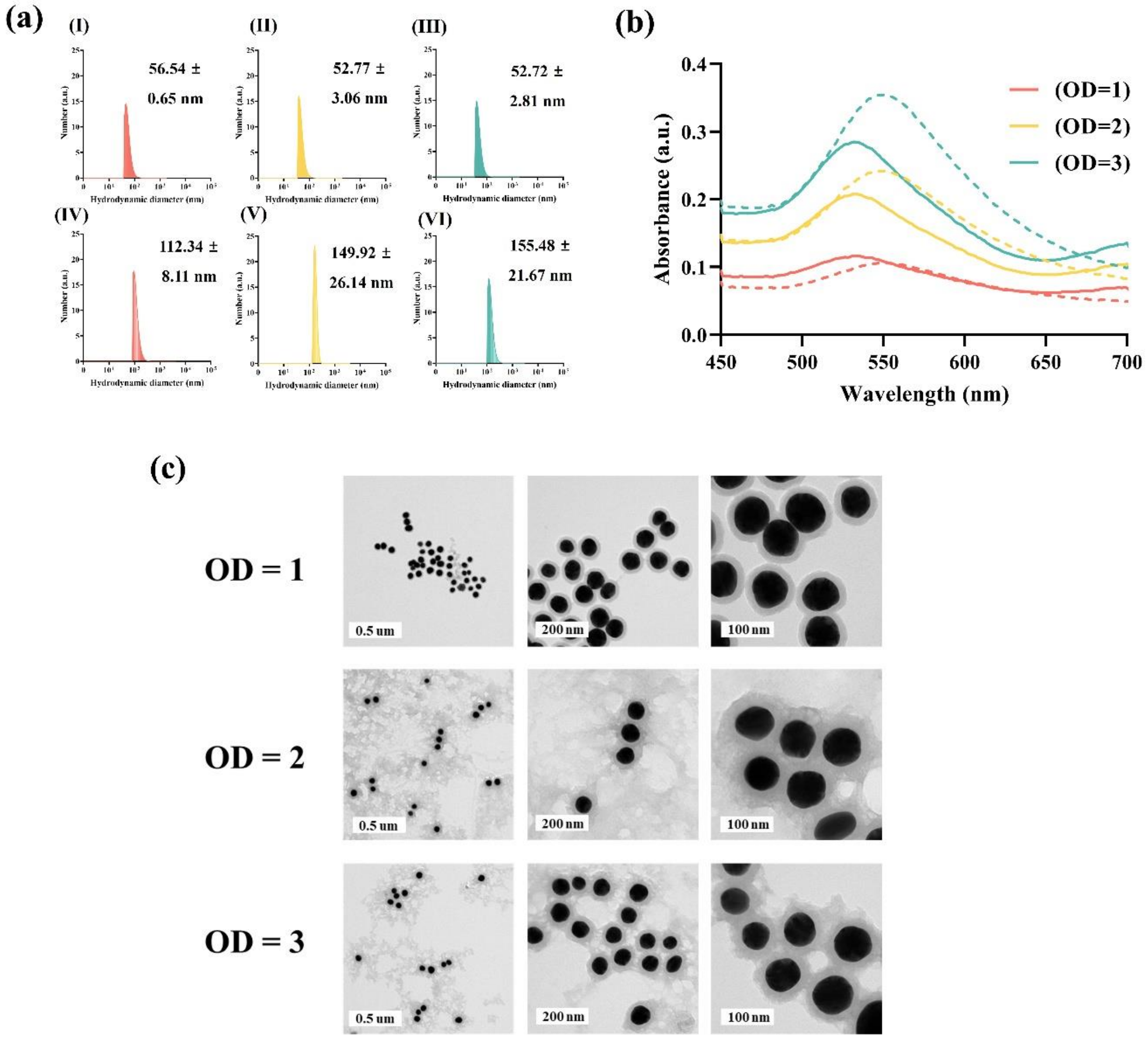

Figure S 1 Analysis of DLS size distribution, UV-vis absorption spectra, and TEM images of GNP and $Au@SiO_2$ at varying optical densities (OD). (a) DLS size distribution of GNP and $Au@SiO_2$ at different OD values: (I) GNP (OD=1), (II) GNP (OD=2), (III) GNP (OD=3), (IV) $Au@SiO_2$ (OD=1), (V) $Au@SiO_2$ (OD=2), and (VI) $Au@SiO_2$ (OD=3), highlighting size variations induced by silica coating and OD adjustments. (b) UV-vis absorption spectra of GNP and $Au@SiO_2$: solid lines represent GNP, while dashed lines represent $Au@SiO_2$, demonstrating shifts in surface plasmon resonance (SPR) as a function of OD. (c) TEM images of $Au@SiO_2$ at different OD values, confirming morphological stability and uniformity in coating across samples.

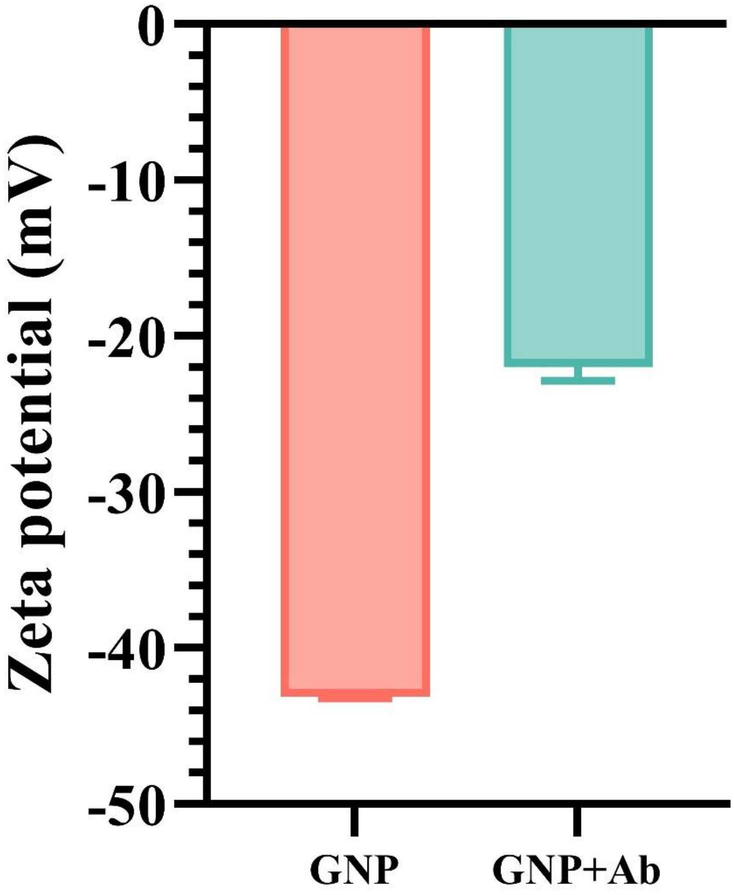

Figure S 2 Analysis of the zeta potential of antibody-conjugated GNP as a control.

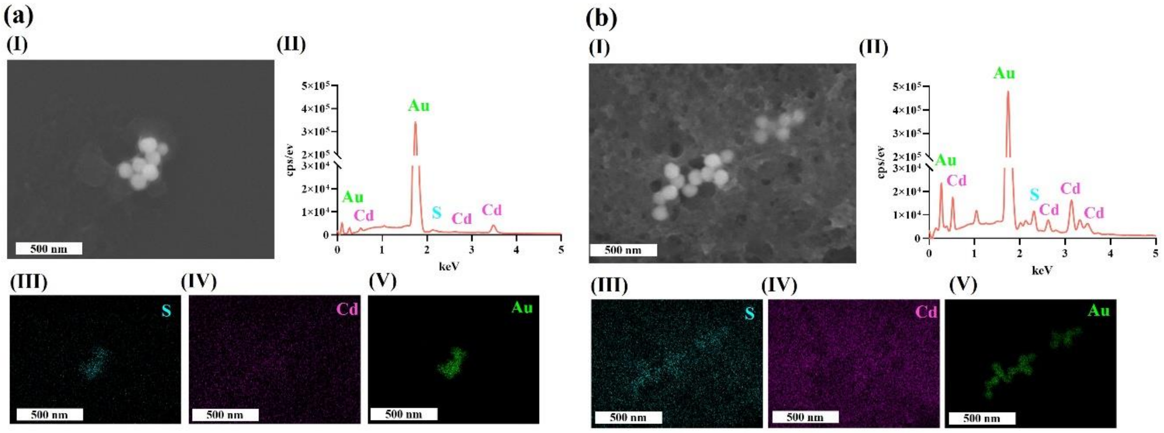

Figure S 3 SEM analysis of BDNF protein impact on CdS QD formation: (a) Absence of BDNF: (I) SEM image showing limited CdS QD formation, (II) elemental composition graph indicating high Au content (68.72%) compared to Cd (26.93%) and S (4.35%), (III–V) elemental mapping images illustrating distributions of S, Cd, and Au. (b) Presence of BDNF: (I) SEM image confirming effective CdS QD formation, (II) elemental composition graph showing a significant decrease in Au content (3.56%) with increased Cd (83.63%) and S (12.82%) proportions, (III–V) elemental mapping images visualizing enhanced distributions of S, Cd, and Au.
